# Lentiviral Mediated Transgenesis by *In Vivo* Manipulation of Spermatogonial Stem Cells

**DOI:** 10.1371/journal.pone.0021975

**Published:** 2011-07-07

**Authors:** Lalit Sehgal, Rahul Thorat, Nileema Khapare, Amitabha Mukhopadhaya, Mugdha Sawant, Sorab N. Dalal

**Affiliations:** KS-215, Advanced Centre for Treatment Research Education and Cancer, Tata Memorial Centre, Kharghar Node, Navi Mumbai, India; Brigham and Women's Hospital, United States of America

## Abstract

This report describes a technique for the generation of transgenic mice by *in vivo* manipulation of spermatogonial stem cells with a high rate of success. Spermatogonial stem cells (SSCs) in pre-pubescent animals were infected *in vivo* with recombinant lentiviruses expressing EGFP-f and mated with normal females. All male pre-founder mice produced transgenic pups with an overall success rate of over 60%. The transgene was heritable and the pre-founder mice could be used in multiple mating experiments. This technology could be used to perform overexpression/knockdown screens in vivo using bar-coded lentiviruses, thus permitting the design of genetic screens in the mouse. Further, this technology could be adapted to other laboratory animals resulting in the generation of model systems that closely approximate human development and disease.

## Introduction

The generation of genetically modified mice has spurred great advances in our understanding of various aspects of growth and development. Multiple technologies have used either injection into a one celled embryo followed by implantation into a pseudo pregnant mother [1], or used stem cell aggregation techniques to generate either knockout [2] or knockdown mice [3]. These experiments are expensive, labor-intensive, time-consuming and require several female donors.

Spermatogonial stem cells are responsible for the production of spermatozoa [4] and are an appropriate target for germline modification [5]. Nagano et al. have generated transgenic mice by infecting spermatogonial stem cells *in vitro* with recombinant retroviruses followed by xenogenic transplantation of the cells into the testes of a male mouse [6]. In some cases the recipient mice were unreceptive to the donor spermatogonial cells [6] and the overall success rate was rather low. Similarly, *in vivo* transduction of testicular germ cells with retroviral constructs carrying a lacZ gene, resulted in a poor success rate of 2.8% [7]. The low success rates post implantation, however precluded these from replacing embryonic injection. Similar experiments using lentiviruses, resulted in better success rates [8]. Other methods employed in the recent past have infected fertilized eggs in vitro with recombinant lentiviruses, followed by implantation of the embryo into pseudopregnant females [9]. While these methods provided better success rates, the implantation experiments are technically cumbersome and require several female donors. A recent report has also described the generation of recombinant spermatozoa in organotypic cultures that could be used to generate transgenic mice [10]. While this method generated transgenic animals at high efficiency, generating organotypic cultures is not trivial and is not performed in most laboratories. Recently, a report has described the generation of transgenic mice after electroporation of DNA into the testes of an adult mouse. 16 of 17 fore founder mice generated in that study were able to sire transgenic pups. However, the report did not mention the rate of transgenesis in the pups [11].

This report describes a new cost effective, faster technique with a high success rate for generation of transgenic mice by *in vivo* viral transduction of the EGFP-f transgene into undifferentiated spermatogonia. These fore-founder male mice were mated with wild type female mice to generate transgenic pups. The rate of transgenesis was greater than 60% and the transgene was inherited in the germ line. This rapid protocol for the generation of transgenic mice could be used to design and perform over-expressor/knockdown screens in the whole animal, thus enhancing our knowledge of disease and development.

## Results

**Figure 1:**
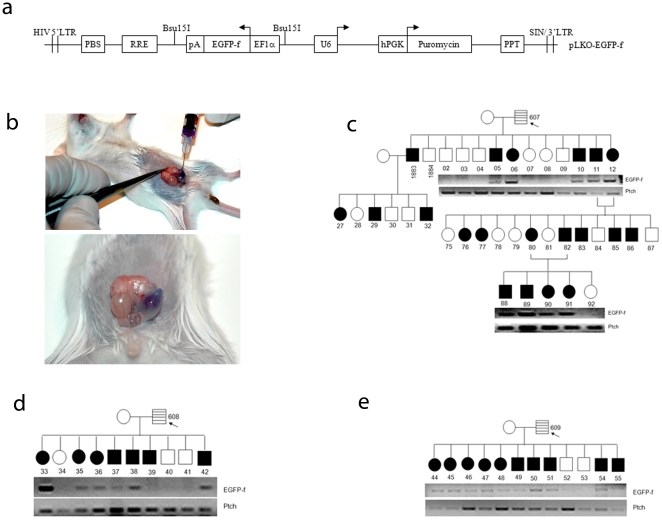
Generation of transgenic mice. **A**. Design of the vector used for generating transgenic mice. **B**. Injection of recombinant lentiviruses into mouse testis. **C–E**. Pedigree analysis for pre-founder mice 607 (c), 608 (d) and 609 (e) showing germline transmission of the transgene. Individual mice were assigned numbers for further experiments. Genomic DNA amplification using primers for EGFP-f or patch (as a loading control) are shown.

Recombinant lentiviruses expressing EGFP-f (EGFP tagged with a farnesylation signal) were injected into the intertubular spaces of the testis targeting undifferentiated spermatogonia present in the seminiferous tubules. Injection into the intertubular space allows the lentivirus to infect undifferentiated spermatogonial cells located at the basement of the seminiferous tubules [12]. Previous results had indicated that a lentivirus vector in which cDNA's were downstream of the CMV promoter was not effective in achieving lentiviral transduction and expression in germ line stem cells [13]. Therefore an expression cassette containing the elongation factor1α (EF1-α) promoter driving the expression of EGFP-f, followed by SV40 poly-adenylation sequence was assembled in pBSK. The assembled cassette was further sub cloned in pLKO.1 [14] to generate a vector that could express both EGFP-f and an shRNA downstream of the U6 promoter (Figure 1A).

5–10 µl of recombinant EGFP-f expressing lentiviruses (5×10^6^ TU/ml) was injected into the intertubular spaces of the testis of 28 day old Crl:CFW(SW) male mice (Figure 1B). These male mice, referred to as pre-founder mice, were mated with wild type females of the same strain 35 days post infection. To determine whether the progeny from this cross carried the transgene, EGFP-f was amplified from genomic DNA isolated from the progeny. An amplification for the patch gene served as a loading control (Figure 1C–E). Transgenic pups were generated from mating experiments with three independently derived pre-founder mice at an overall rate greater than 60% (Figure 1C–E, Table 1), a rate that is much higher than previously reported with conventional transgenic protocols or with retroviral infection of spermatogonial stem cells *in vitro* or *in vivo* [6], [7]. All mice showed amplification for the patch gene, which served as a loading control and also demonstrated that the band for EGFP-f was specific. Further, WT mice did not show any product for the EGFP-f transgene (data not shown). Inbreeding of transgene positive pups from the mice showed an increased incidence of transgene positivity in the F1 generation and an even higher incidence in the F2 generation (Figure 1C). Further, a transgene positive male mouse, 1883, was out bred with a wild type female mouse of the same strain. Three of the six pups from this cross showed the presence of the transgene (Figure 1C). These results suggest that inheritance of the transgene is stable and that fertility of the transgenic mice is not compromised.

**Table 1:** Percentage of EGFP-f positive pups obtained from individual matings with three different pre-founder mice.

Pre-founder mice	Founder Mice per litter	Founder mice per litter positive for EGFP-f
607	8	1
607	11	9
607	6	4
Total	25	14
Success rate		56%
608	8	5
608	6	4
608	8	4
Total	22	13
Success rate		59%
609	8	4
609	12	10
609	13	7
Total	33	22
Success rate		66%
Grand Total	**80**	**49**
	**Total success rate**	**61.25%**

The pre-founder mice were mated with multiple WT female mice and the frequency of EGFP-f positive pups determined after each mating. Note that an overall success rate of greater than 60% was obtained in these matings.

**Figure 2:**
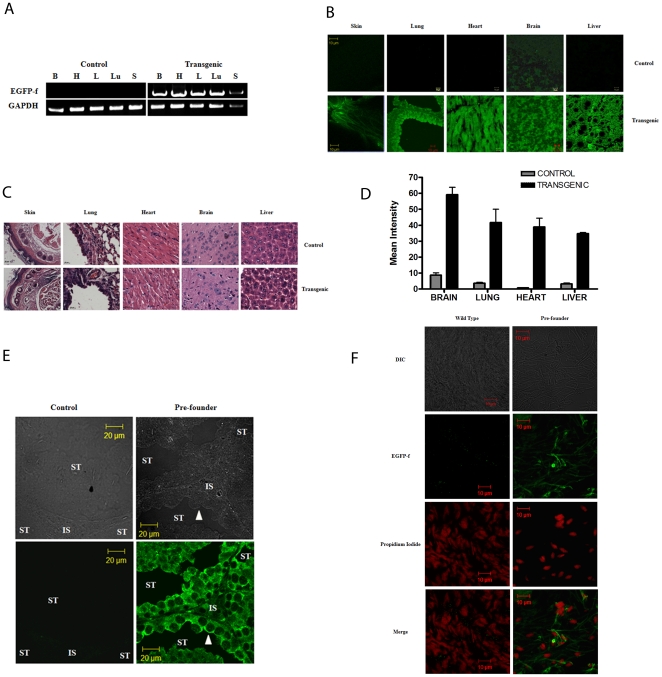
EGFP-f expression in various tissues of founder mice. **A**. Multiple tissue sections from control and transgenic littermates were analyzed for the expression of EGFP-f mRNA by RT-PCR analysis. Control mice did not show the presence of the EGFP-f mRNA but transgenic mice showed the expression of the EGFP-f mRNA in multiple tissues (H = heart, B = brain, L = liver, Lu = lung and S = skin). An RT-PCR was performed for GAPDH as a loading control. **B**. Multiple tissue sections from control and transgenic littermates were analyzed for the expression of the EGFP-f transgene by confocal microscopy. Original magnification is 630×. Scale bars are shown in the figures. **C**. Hematoxylin and eosin stained sections from wild type or transgenic mice. Original magnification is 630×. Scale bars are shown in the figures. **D**. The mean fluorescence intensity from control and transgenic sections is on the Y-axis and the different tissues are represented on the X-axis. The error bars represent the standard deviation for sections derived from at least three different mice. **E**. Testis sections from control mice or pre-founder mice were stained with antibodies to GFP followed by immunflourescence analysis and confocal microcospy. The top panel shows the DIC image of the fluorescence field. Note that the pre-founder mice show high levels of EGFP-f expression in both cells in the interstitial spaces and in spermatogonial stem cells (filled arrow) that line the basement membrane of the seminiferous tubules. Original magnification is 630× and scale bars are indicated in the figures. ST = seminiferous tubule and IS = interstitial spaces. **F**. Epididymis sections from control mice or pre-founder mice were stained with antibodies to GFP (green) and propidium iodide (red) to stain DNA, followed by immunflourescence analysis and confocal microcospy. The top panel shows the DIC image of the fluorescence field and the bottom panel shows the merged image. Note that the pre-founder mice show high levels of EGFP-f expression in the sperm tails in the profounder mouse but not in WT mice. Original magnification is 630× and scale bars are indicated in the figures.

To determine whether the transgene was expressed in multiple tissues, tissues from organs of an F1 GFP positive mouse and a GFP negative litter mate were analyzed for the presence of the EGFP-f transgene by Reverse Transcriptase PCR (RT-PCR) and fluorescence microscopy. As shown in Figure 2A, the EGFP-f transgene positive animal showed the presence of the EGFP-f message as compared to RNA prepared from tissue sections derived from the negative litter mate control. A RT-PCR analysis for the housekeeping gene, GAPDH, served as a loading control. Similarly, tissue sections from the transgenic mice showed green fluorescence in multiple tissues, when compared to the control mice (Figure 2B). An analysis of hematoxylin and eosin stained sections of different tissues indicate no change in the morphology of tissue from transgenic mice as compared to control mice (Figure 2C). The mean intensity fluorescence obtained by three different section of the same tissue of transgenic mice is much higher when compared to control mice (Figure 2D).

To demonstrate that the spermatogonial stem cells had been infected in the pre-founder mice, the mice were sacrificed after multiple breeding experiments and tissue sections prepared from the testis of the pre-founder and control mice. The tissue sections were stained with antibodies to GFP. As shown in Figure 2E, cells in the interstitial spaces and the spermatogonial stem cells showed staining for GFP in pre-founder but not in control mice. An immunofluoresence analysis on sections from the epididymis followed by confocal microscopy demonstrated that mature sperm also stained positively for GFP in profounder mice, whereas sections from wild type mice did not show any signal for GFP (Figure 2F). The GFP staining mostly localized to the tail region and was mostly excluded from the head region.

**Figure 3:**
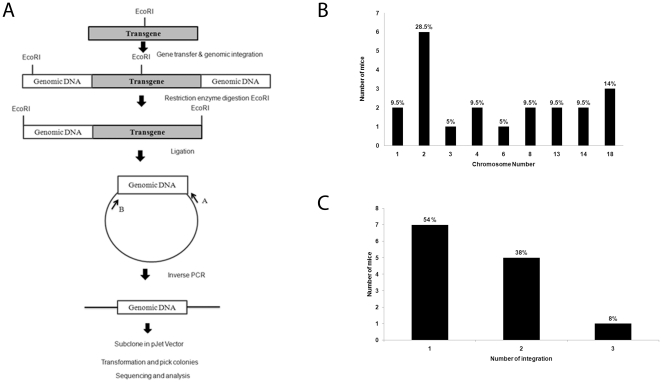
Analysis of integration events in the EGFP-f transgenic mice. **A**. Protocol for the identification of integration sites. **B**. Graph showing the frequency of integration events observed in the transgenic animals. Chromosome number is on the X-axis and the number of mice showing integration in the respective chromosomes is on the Y-axis. The number above each bar in the graph indicates the percentage of total integration events analyzed on each chromosome. **C**. Graph showing the number of integration events in each mouse. No of integration events is on the X-axis and number of mice on the Y-axis. The number above each bar indicates the percentage of mice with one, two or three integration events.

The pre-founder mice were able to sire transgenic pups for over a year after infection with the recombinant lentivirus indicating that the transgene was integrated in the spermatogonial cells. To demonstrate that expression of the transgene was independent of the site of integration, integration sites were mapped in founder mice using a variant of a protocol developed by Schroder et al. [15] as summarized in Figure 3A. The integration events to different sites on chromosomes were represented graphically as described earlier [16]. In addition, we have listed the percentage of total integration events on each chromosome in Figure 3B. Most of the integration events were observed in three different sites on chromosome two. This resulted in the maximum number of mice having integrants in chromosome two. The sites of integration were sequenced and the distribution among the different chromosomes mapped as shown in Figure 3B and Table 2. These results show that expression of the transgene was independent of the site of integration. Further, the number of integration events in each transgenic mouse was determined. It was observed that most mice had either one or two integration events (Figure 3C). The percentage of mice having either one, two or three integration events is also listed in Figure 3C. This suggests that at the virus titer used in these experiments most spermatogonial stem cells develop either one or two integration events.

**Table 2:** Summary of the integration events observed in the different transgenic animals.

Chromosome number	Accession Number	Number of mice showing integrations	Identity of mice showing integrations
1	NT_39170.7	3	6 &,12
2	NW_001030712.1 NW_001030694.1 NW_001030686.1	9	11,12,33,35,45 &51
3	NT_039207 NT_162143	2	38
4	NW_001030747	2	49 & 54
6	NW_039353	1	33
8	NT_078575.6	3	11 & 45
13	NT_039578	2	6 & 38
14	NW001030543	2	42 & 37
18	NW_001030631 NW_001030635.1	3	12 & 52

The chromosome number is shown in column one while the number of the individual mice with an integration in these chromosomes is shown in column 4. The integration events in each chromosome are shown in the third column of this table with the appropriate accession number in column 2. As can be seen from the table, multiple mice show different combinations of integration events suggesting that transgene expression is independent of integration site. The number of assigned to the mice in this table is similar to the number reported in Figure 1.

## Discussion

The experiments in this report describe the development of a simple, cost effective, and efficient technique for the generation of transgenic mice by *in vivo* transduction of the desired gene/shRNA construct into undifferentiated spermatogonia of the testis. This technology does not compromise the fertility of the off-spring, resulting in germline transmission of the transgene, using a limited number of animals.

Previously published reports have demonstrated that lentiviral mediated transgenesis results is relatively stable and can be inherited in the germline [9], [17], [18], [19]. Thus, the use of lentiviruses as a vector delivery system to generate transgenic animals does not compromise inheritance or the development of the animal, results that are consistent with those obtained in this report. Further, previous protocols that modify spermatogonial stem cells, either in vitro or in vivo, used retroviruses and yielded transgenic pups at very low efficiencies [6], [7]. Previously, male rat germ cells were transduced in culture using HIV-1 based lentiviral vectors, which were then transplanted into the testis of wild type rats [8]. The rate of colonization of the recombinant germ cells in wild type testis was low (33%) and only one of three male mice was fertile. The founder mice in these experiments produced founder pups at a rate of 30% [8]. The processes reported in this paper result in a very high rate of transgenesis, with all the animals being able to sire transgenic pups, leading to the rapid generation of multiple transgenic pups with different integration events allowing the generation of multiple transgenic lines.

Recently, organotypic testis cultures have been generated that allow the production of recombinant sperm, which could be used to generate transgenic animals [10]. While this allows the generation of recombinant sperm without the generation of profounder animals, the process can be technically challenging, especially for laboratories not used to establishing and propagating organotypic cultures. The procedures described in this report are easily performed and require a small simple surgery. Although, the rate of transgenesis reported here may not be better than the conventional lentiviral transgenesis method [9], the technique is cost effective, simple and faster to perform. Further, infecting the spermatogonial stem cells in vivo allows the repeated use of the pre-founder, eliminating the necessity to repeatedly infect embryos or organotypic cultures with lentiviruses. Further, as demonstrated here the pre-founder mice can be mated multiple times, resulting the generation of a number of transgenic pups. As multiple transgenic lines are required to rule out integration site specific events, the generation of multiple lines described herein results in a quick analysis of the phenotype and allows experiments to proceed at a rapid pace.

Several groups have reported that knockdown mice replicate the phenotypes observed in knockout mice [3], [20], [21]. While this greatly enhances the ability of scientists to study the consequences of gene depletion in the whole animal, the use of implantation strategies did not permit the use of genetic screens using lentivirus driven bar-coded shRNA's, as performed in cell lines in culture [22], [23]. The protocol described in this paper now permits the use of such genetic screens in the whole animal to understand the contribution of various gene products to growth, development and disease. Screens using tissue specific production of miRNAs [24], or by using either temporal or tissue specific Cre based inducible systems [20], [21] can also be designed to examine the effects of knockdown/overexpression of a gene product in a particular tissue type. As the experiments described in this report have shown, most of the mice have one or two integration events. Therefore, it should be possible to design genetic screens without worrying about the complications that could ensue from multiple integration events in the same animal. The number of integration events could be increased or decreased by altering the titer of the virus used for injection; however, lowering titer might hamper efficiency of generation of transgenic mice in the F1 generation. The design of the construct used in this report also permits that an in vivo rescue experiment could be performed using one lentiviral construct that expresses both the shRNA and the resistant cDNA from different promoters, thus eliminating concern about off target effects of the shRNA construct. Finally, the procedure could be extended to other animals, especially non-human primates, resulting in a significant advancement in transgenic research and the use of models that are closer to human subjects to model human disease.

## Materials and Methods

### Animals

Swiss mice Crl:CFW(SW) were bred and maintained in the laboratory animal facility of ACTREC. Maintenance of the animal facility is as per the national guidelines provided by the Committee for the Purpose of Control and Supervision of the Experiments on Animals (CPCSEA), Ministry of Environment and Forest, Government of India. The animals were housed in a controlled environment with the temperature and relative humidity being maintained at 23±2°C and 40–70% respectively and a day night cycle of 12 hrs each (7:00 to 19:00 light; 19:00 to 7:00 dark). The animals were received an autoclaved balanced diet prepared in-house as per the standard formula and sterile water *ad libitum*. Mice were housed in the Individually Ventilated Cage (IVC) system (M/S Citizen, India) provided with autoclaved rice husk bedding material available locally. Protocols for the experiments were approved by the Institutional Animal Ethics Committee (IAEC) of ACTREC. The animal study proposal number is 11/2008 dated August 19, 2008.

### Generation of Lentiviral vectors

The EGFP-f (Farnesylated EGFP) expression cassette was assembled in pBSK(-) (Stratagene). The sequences of all the oligonucleotides used for gene amplification are shown in Table 3. The EF1α promoter was amplified from pEF6MycHisA (Invitrogen) and cloned in pBSK digested with HindIII and EcoRI (NEB). Subsequently, the EGFP-f gene was amplified and cloned downstream of the EF1α promoter as a EcoRI and BamHI fragment. The resulting vector was digested with BamHI and NotI and the SV-40 Poly A signal, amplified from pEF6MycHisA, was inserted using these restriction sites. A restriction site for BsuI5I (Fermentas) was introduced upstream of the NotI restriction site. The EGFP-F expression cassette was excised from pBSK (-) vector with BsuI5I and cloned in pLKO.1 to generate pLKO.1 EGFP-f.

**Table 3:** List of oligonucleotides used for PCR reactions.

Name of oligonucleotide	Sequence
EF1α Forward	AAGCTTGGAATTGGCTCCGGTGCCCGTC
EF1α Reverse	GAATTCCCTCACGACACCTGAAATGG
EGFP-f Forward	GAATTCCACCATGGTGAGCAAG
EGFP-f Reverse	GGATCCCCTCAGGAGAGCACACACTT
PolyA Forward	GGATCCCCATACCACATTTGTAGAGG
PolyA Reverse	GCGGCCGCATCGATACATTGATGAGTTTGGACAAACCAC
Ptch Forward	CTGCGGCAAGTTTTTGGTTG
Ptch Reverse	AGGGCTTCTCGTTGGCTACAAG
IPCR A	CGGCGCGCCCTGCTGAGC
IPCR B	GGCCACCGTCGGCGTCTCGCCCG
GAPDH Forward	GGCTGCCCAGAACATCAT
GAPDH Reverse	CGGACACATTGGGGGTAG

### Virus Production

The lentiviral vector pLKO.1 EGFP-f was co-transfected with the Vira Power packaging mix into 293-T cells and viruses were harvested as per the manufacturers instructions (Invitrogen). The lentiviruses were concentrated by centrifuging the tissue culture supernatant at 35000× g in a swinging bucket rotor for 45 minutes at 4°C. The viral pellet was then dissolved in 1/100^th^ volume of Dulbeco's PBS (Invitrogen). The viral titre was determined using flow cytometry for EGFP-f as per the manufacturers protocol (Invitrogen).

### Injection of viral particles and generation of transgenic mice

28 day old Crl:CFW(SW) male mice were anesthetized by intra-peritoneal injection of Avertin (Sigma) (2,2 tribromo-ethanol and t-amyl alcohol at the rate of 0.015 ml/gm body weight). Hair was removed from the inguinal area of mice and the surgical site was cleaned using betadiene. Anterior to the penis, a single midline cutaneous incision of approx. 1–1.5 cm length was made using sterile surgical scissors under aseptic conditions. After making the incision in the muscles, the testes were removed from the scrotal sac with a curved sterile forceps through the incision by gently pulling the dorsal fat pad associated with the testis. A solution of lentiviruses resuspended in dPBS (Invitrogen) containing trypan blue (0.04%) was injected slowly into the inter-tubular space of one testis using a 30 gauge needle. The untransduced testis was vasectomised. The animals were kept on thermal plate until they recovered from the surgery to avoid hypothermia. The pre-founder male mice were co-habitated with wild-type females 35 days post-transduction and the pups generated were analyzed for the presence of the EGFP-f transgene.

The pre-founder male mice were mated with wild-type females (ratio 1∶2). Pups born were analyzed by PCR using genomic DNA obtained from their tail tip and those positive for the transgene were considered as F1 generation of transgenic animals. F2 generation of mice were produced either by breeding transgene positive animals with wild-type Crl:CFW(SW) mice or by breeding two mice positive for the presence of the transgene. The pups from these matings were analyzed for the presence of the transgene as described.

### Isolation of Genomic DNA (gDNA)

Tail biopsies (∼3 mm) from 3 weeks old pups sired by pre-founder males were taken and lysed for 16 h at 55°C in high salt digestion buffer containing 50 mM Tris HCl, 1% SDS, 100 mM NaCl, 100 mM EDTA and 1200 µg/ml Proteinase K (Jackson Laboratories). The lysate was processed for isolation of DNA using phenol-chloroform extraction followed by ethanol precipitation.

### Polymerase Chain Reactions and Reverse Transcriptase PCR

The genomic DNA was subjected for PCR analysis using transgene-specific primers (Table 3). Every PCR reaction set had three controls. The pLKO.EGFP-f plasmid was used as a template for a positive control, the genomic DNA obtained from WT mice was used as a negative control and amplification of the endogenous patch gene was used as a loading control. RNA was prepared from tissues using the Qiagen RNeasy kit as per the manufacturers protocol and RT-PCR was conducted using RevertAid™ First Strand cDNA synthesis Kit (NEB) according to manufacturer's protocol.

### Fluorescence and Immunofluorescence microscopy

5 µm cryosections of multiple tissues were observed by confocal microscopy to detect the presence of EGFP-f. Confocal images were obtained by using a LSM 510 Meta Carl Zeiss Confocal system with an Argon 488 nm and Helium/Neon 543 nm lasers. All images were obtained using an Axio Observer Z.1 microscope (numerical aperture [NA] 1.4) at a magnification of ×63. 5 µm cryosections of testis were deparafinzed and treated with 1% sodium borohydrate at room temperature to reduce auto-fluoresence of tissues. Blocking was performed with 1%BSA and tissues were further immunostained using GFP monoclonal antibody 1∶50 (clonetech) overnight to detect the presence of EGFP-f. The next day, the sections were washed and incubated with the secondary antibody (Goat Anti mouse Alexa fluor 568 1∶200 Invitrogen). Prpodium iodide was used to stain sperm DNA. Confocal images were obtained as above. The image was further analyzed using LSM 510 image browser and pseudoclour (green) was used for representing the EGFP-f expression.

### Inverse PCR and mapping of integration sites

Lentiviral integration sites were identified by modifying the ligation-mediated PCR method as described in Figure 2D [15]. In brief, 5 µg genomic DNA was digested with EcoRI for 4 h and purified using the Qia-Quick PCR purification kit (Qiagen). The purified DNA was then self-ligated with T4 ligase (NEB) at 4°C overnight. The circular DNA was amplified using a specific primer pair (Table 3). The amplified products were cloned into the pJet1.2/Blunt vector (Fermentas) followed by sequencing on a 3100 Avant Genetic Analyser. The nucleotide sequences were aligned and examined to confirm the presence of the expected known transgene sequence and to identify the flanking sequence which would be derived from the site of insertion. These flanking sequences were matched with the mouse genome using a stringency of >90% over at least 100 bp. The insertion site sequence was analyzed using the Basic Local Alignment Search Tool (BLAST) to determine the chromosomal location of the transgene.
